# Effects of Trace Elements on the Fatty Acid Composition in Danubian Fish Species

**DOI:** 10.3390/ani14060954

**Published:** 2024-03-19

**Authors:** Katarina Jovičić, Vesna Djikanović, Isidora Santrač, Sanja Živković, Milena Dimitrijević, Jelena S. Vranković

**Affiliations:** 1 Department of Hydroecology and Water Protection, Institute for Biological Research “Siniša Stanković”—National Institute of Republic of Serbia, University of Belgrade, Bulevar Despota Stefana 142, 11008 Belgrade, Serbia; djiki@ibiss.bg.ac.rs (V.D.); jeca.s@ibiss.bg.ac.rs (J.S.V.); 2 Life Sciences Department, Institute for Multidisciplinary Research, University of Belgrade, Kneza Višeslava 1, 11000 Belgrade, Serbia; isantrac@imsi.bg.ac.rs (I.S.); milena.dimitrijevic@imsi.bg.ac.rs (M.D.); 3 Department of Physical Chemistry, “Vinča” Institute of Nuclear Sciences—National Institute of the Republic of Serbia, University of Belgrade, 11351 Belgrade, Serbia; sanjaz@vin.bg.ac.rs

**Keywords:** roach, white bream, lipids, inorganic chemicals, Danube

## Abstract

**Simple Summary:**

Anthropogenic pollution poses a major threat to aquatic ecosystems, which can lead to their degradation. The accumulation and toxicity of metals and trace elements in fish leads to physiological and chemical changes in the fish body. In this study, we investigated the effects of bioaccumulation of metals and trace elements on freshwater fish fatty acid profiles at two different sites before and after the discharge of untreated municipal wastewater in two fish species with different diet habits. Although the concentrations of toxic elements were below the maximum levels proposed by the EU and the Republic of Serbia, this study showed statistically significant correlations between the presence of certain elements and the fatty acid (FA) profile in fish muscle. Lower concentrations of polyunsaturated FA in fish sampled after the discharge of untreated municipal wastewater were detected.

**Abstract:**

In this study, the concentrations of metals and trace elements (As, Cd, Co, Cr, Cu, Hg, Ni, Pb and Zn) were determined in the muscle tissue of adult roach and white bream at two different sites in the Belgrade section of the Danube. Twenty-six fatty acids, consisting of nine saturated FA (SFAs), seven monosaturated FA (MUFAs) and ten polysaturated FA (PUFAs), were identified. The analysis of the concentration of metals and trace elements of the roach and white bream showed species-specific differences in their bioaccumulation. Four of all elements analyzed (As, Hg, Ni and Pb) correlated significantly with the changes in FA profiles in fish from both sampling sites, with the exception of Cu, which correlated with the FA profile at the site before, and Zn, whose concentration influenced the FA profile at the site after wastewater discharges. The lower PUFA content in the fish from a site under higher environment pressure could indicate that the fish are stressed. The results suggest that changes in lipid composition may be one of the protective mechanisms of cells to cope with anthropogenic stressors.

## 1. Introduction

The rapid development of industry and agriculture has led to increased levels of inorganic (e.g., metals and trace elements) and organic chemicals in the aquatic environment. The discharge of wastewater enriched with these chemicals can lead to pollution of the aquatic environment and thus affect biological communities [1].

In Serbia, the lack of wastewater treatment and practices leads to pollution of water resources with metals and other pollutants, which damages the aquatic ecosystem. Belgrade is the most important industrial area in Serbia, and the largest industrial capacities are located mainly on the banks of the Danube. The main problem related to river pollution in Belgrade is the discharge of wastewater without prior treatment [2].

Metals pose a particular threat to the environment due to their high potential for bioaccumulation and biomagnification in living organisms [3]. The accumulation and toxicity of metals in fish is multidirectional and leads to physiological and chemical changes in the body of fish. Metals can stimulate an excessive formation of reactive oxygen species and disrupt the balance of oxidation–reduction reactions, resulting in structural damage to lipids (primarily polyunsaturated fatty acid (PUFAs)), proteins, and DNA [4]. The presence of contaminants such as metals can greatly affect fatty acid (FA) composition through changes in the relative abundance of individual fatty acids [5,6,7,8,9,10]. Hence, the study of the lipid profile also serves as a biomarker of fish health [11]. Fat content and composition can vary greatly between different fish species and even within the same species when reared under different conditions [12]. Fish muscle is an important source of FA, especially long-chain PUFAs [1,13]. Among PUFAs, omega-3 FA are important because of their involvement in several physiological processes and their nutritional significance [14].

The extent of the bioaccumulation of metals in fish tissues is influenced by abiotic and biotic factors, such as the various organic and inorganic pollutants in the water, water temperature, oxygen concentration, and pH, and biotic factors, such as sex, age, body weight and the physiological condition of the fish [15,16]. Dietary habits can have a major influence on the accumulation of toxic elements in various fish tissues [15,17,18,19]. Due to their ability to biomagnify, predatory fish accumulate higher amounts of metals compared to species that are at a lower trophic level [20]. Metal accumulation in fish depends on pollution and can be different for different fish species living in the same water body [21].

Recently, non-traditional biochemical biomarkers, namely the FA profiles of organisms, have been tested and proposed as reliable indicators of pollution levels [10,22,23,24]. Changes in lipid metabolism and profiles of FA have been used to better understand how pollution affects organisms in aquatic food webs and as an integrative biochemical response to contaminant exposure and accumulation in aquatic organisms [22] involving several studies with metals [25,26], making them promising biomarkers for contaminant exposure assessment. Previous studies with metals support the use of lipid metabolism and FA profiles as valuable biomarkers to assess contaminant exposure [22,25,26], while most studies have focused exclusively on the accumulation of metals or on analyzing the composition of FA [27,28,29,30].

For the purposes of this study, two freshwater fish species with different feeding habits were selected. The roach *Rutilus rutilus* (Linnaeus, 1758) is omnivorous and opportunistic, feeds on plants, a variety of small invertebrates (insects, crustaceans, and mollusks), and small fish. The diet of the roach is diverse and adaptable, allowing it to survive in a number of different water bodies, making this species very widespread. The white bream *Blicca bjoerkna* (Linnaeus, 1758) is a benthivorous fish that feeds on benthic invertebrates such as insect larvae, mollusks, crustaceans. It also feeds on aquatic vegetation such as algae, plant debris, and macrophyte seeds, especially during the warmer months of the year. Overall, the diet of the white bream is variable and influenced by season, location, and food availability [31]. In addition, these two fish species have a different preference for micro-habitats. The roach prefers lentic habitats with low water velocity, while the white bream is common in the head of the river with fast currents [31]. In this study, we wanted to investigate whether there is a relationship between the presence of inorganic elements and changes in the FA profile in fish muscle sampled from two localities with different pollution pressures.

## 2. Materials and Methods

### 2.1. Study Site and Sample Collection

**Figure 1:**
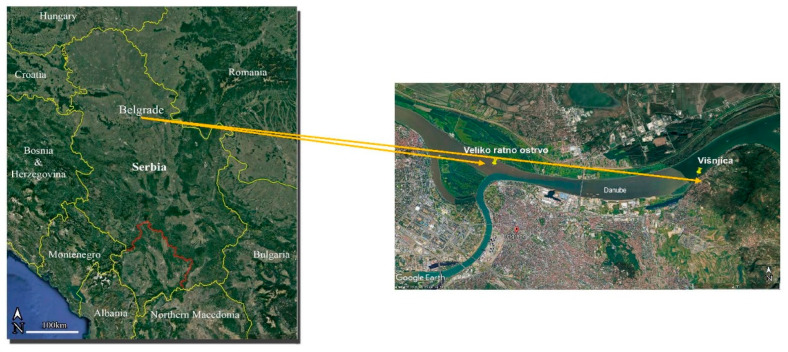
Map of two sampling sites: Veliko Ratno Ostrvo (VRO) and Višnjica (VIS) (source: Google Earth).

All specimens of roach and white bream were collected by professional fishermen in April 2021. This study was conducted at the Danube sites Višnjica (VIS, 1162 river kilometers), which is exposed to the discharge of the largest wastewater collector of the city of Belgrade [32], and Veliko Ratno Ostrvo (VRO, 1170 river kilometers) at the confluence of the Danube and Sava rivers, upstream of the collector (Figure 1). To avoid sex, age and size differences, 16 adult male roaches and 16 adult male white breams were selected. The total body length and total body weight of the fish were measured (see Supplementary Table S1). Muscle tissue samples were taken separately from each fish of both species for elemental and FA analysis. The samples were washed with distilled water and stored at −20 °C prior to analysis.

### 2.2. Elemental Accumulation Analysis

Analytical portions of approximately 0.5 g (wet weight) were accurately weighed and then processed in a microwave-assisted digestion system. Samples were mineralized by adding 9 mL of 65% HNO_3_ and 1 mL of 30% H_2_O_2_ (Merck, Darmstadt, Germany). Microwave-assisted digestion was performed in ETHOS EASY Advanced Microwave Digestion System 230 V/50 Hz, Milestone, Italy. After cooling, the digested samples were diluted with distilled water to a total volume of 25 mL. To assess the possible presence of trace elements in the reagents and the carry-over effect of the digestion vessels, five reagent blanks were prepared during sample preparation, one per each session, according to the described procedure. These samples were analyzed in each analytical batch. All solutions were stored in polyethylene bottles until the trace elements (As, Cd, Co, Cr, Cu, Hg, Ni, Pb, and Zn) were measured using inductively coupled plasma optical emission spectrometry (ICP-OES) via the Thermo Scientific™ iCAP™ 7400 ICP-OES (see Supplementary Table S2). The single-element mercury (Hg) calibration standard (1000 mg/mL) and the multi-element standard (100 mg/mL 21 components) purchased from CPAChem, Bulgaria, were used to prepare the calibration standards for the performed ICP-OES analysis. All measurements were performed in triplicate. Thermo Scientific Qtegra Intelligent Scientific Data Solution (ISDS) software was used for data acquisition and processing. The detection limits were 1 ppb for Ni, Cd, Cr, Co and Hg, 2 ppb for As, and 30 ppb for Zn.

### 2.3. Fatty Acid Profile Analysis

Samples of muscle tissue, about 25 g per sample, were measured. Before homogenization, an appropriate amount of anhydrous Na_2_SO_4_ and PetrolEtra was added to each sample (10 g of anhydrous Na_2_SO_4_ and 15 mL of PetrolEtra were added to 10 g of tissue). Then, the tissue was homogenized with a Politron, Kinematika (CH -6010 Kriens, Lucerne, Switzerland) for 5 min at speed 5. The supernatant was filtered through filter paper into a flask and evaporated to dryness on a vacuum evaporator from IKA, Germany (IKA RV 10), at 40 °C and 150 rpm.

The analysis of the fatty acid methyl ester profile (FAME) included transesterification by acid methanolysis and gas chromatography–mass spectrometry (GC-MS). The extracted lipids (30 mg) were dissolved in 6 mL of methanol with 2–3 drops of concentrated sulfuric acid. The mixture was refluxed at 80 °C for 2 h and then the pH was adjusted to 7 with NaHCO_3_ solution (0.1 g/mL water). The FAMEs were collected using hexane (4 × 6 mL). The hexane layer was collected with a Pasteur pipette and dried with 15 g of anhydrous Na_2_SO_4_ for 15 min. The solution was filtered to remove the desiccant, and the solvent was removed in a rotary evaporator from IKA, Germany (IKA RV 10), at 40 °C with 150 rpm. The FAME extract was dissolved in hexane (5 mg/mL) and filtered through a Nachlon syringe filters 0.22 µm. The analysis was performed using a GC-MS KP2010 plus, equipped with an AOC 5000 injector (Shimadzu, Kyoto, Japan) and a FAME column (Phenomenex, L = 30 m, ID = 0.25 mm, df = 0.50 µm), and using GCMS solution Ver. 2 software (Shimadzu). The samples (1 µL) were injected in split mode (1:30), with the injector temperature set at 250 °C. Mass spectra were obtained in EI mode (±70 eV) in the m/z range 50–500 amu (SCAN) mode. Helium (99.999%) was used as a carrier gas with a flow rate of 1.34 mL/min. The column was heated linearly from 100 °C (hold for 2 min) to 240 °C with a gradient of 3 °C/min and held at 240 °C for 5 min. The ion source temperature was set to 240 °C; the interface temperature was up to 260 °C. Constituents were identified by comparing their mass spectra with those of the NIST05, Wiley8, and FFNSC3 libraries using different browsers and a set of FAME standards in a Supelco^®^ 37-component mixture FAME dissolved in hexane (1 mg/mL). Quantitative data were determined from the GC peak area using the area normalization method (the results obtained are expressed as relative percentages).

### 2.4. Statistical Analysis

The initial assessment of the differences between groups was performed using the Kruskal–Wallis H test, which indicated differences in metal and trace element concentrations between localities (*p* < 0.05 was used as the threshold, STATISTICA 12.0). Differences in tissue metal concentrations between different sites of the same species were estimated using the Mann–Whitney test (*p* < 0.05). Principal component analysis (PCA) was implemented to statistically determine the differences between roach and white bream based on analyzed parameters (FA and metals), and to detect variables that significantly contributed to differences in the analyzed parameters. After examining the fatty acids’ composition and essential metals’ and trace elements’ concentrations in the fish muscle, two PCA models were constructed. Spearman’s rank correlation was performed separately in white bream and roach between the investigated metals and FA with respect to the investigated localities VRO and VIS. Also, the same analyses were performed between body length and the analyzed parameters in white bream and roach, separately.

## 3. Results and Discussion

### 3.1. Metal and Trace Element Concentrations in Fish Muscle

**Figure 2:**
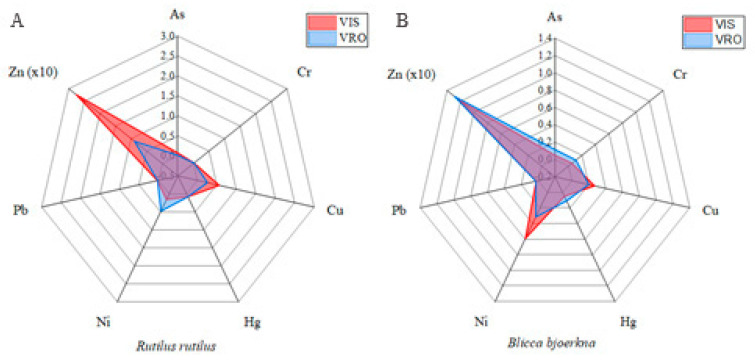
Radar plots presenting concentration of selected metals in roach (**A**) and white bream (**B**) at the Veliko Ratno Ostrvo (VRO) and Višnjica (VIS) localities.

The concentration of nine metals and trace elements in the muscle tissue of roach and white bream was measured. The average concentrations of the analyzed metals and trace elements (As, Cd, Co, Cr, Cu, Hg, Ni, Pb, and Zn) are shown in Table 1 and Figure 2 (radar plots). In the total of 32 fish samples, the highest values were generally measured for Zn, while Cd and Co were below detectable levels. It should be borne in mind that although the concentrations of Cd and Co have been below the detection limit in muscle compared to the measured concentrations of other elements, these metals are toxic in very low concentrations in the tissue itself; so, their effects cannot be ruled out. Comparable consequences have been reported by many researchers in studies for different fish species [32,33,34]. The detected As, Cu, Hg, Pb and Zn concentrations were below the maximum allowable concentrations set by the EU [35] and the Republic of Serbia [36]. In roach, the bioaccumulation of As, Pb and Zn differed significantly (*p* < 0.05) between localities. The muscle had higher levels of As (*p* < 0.05), Cr, Cu, Hg, Pb (*p* < 0.05), and Zn (*p* < 0.05), except for Ni in roach from VIS compared to that from the VRO locality. The opposite trend was observed for the levels of As, Cr, Hg, Pb, and Zn, but there were no significant differences between the levels of all elements measured in white bream muscle at the two sites (Table 1, Figure 2).

The results differ from previous analyses of metal concentrations in fish muscles at the same sampling sites as in this study [37,38,39]. As for the VRO site, the Cr concentrations in fish muscles determined in this study are consistent with the results of Subotić et al. [38,39] (Table 1). The same authors reported much higher concentrations of As, Cu, and Hg in the muscle of common nase *Chondrostoma nasus* (Linnaeus, 1758), Vimba bream *Vimba vimba* (Linnaeus, 1758), sichel *Pelecus cultratus* (Linnaeus, 1758), and ruffe *Gymnocephalus cernua* (Linnaeus, 1758) compared to those found in this study. Lead concentrations in all of the above fish species were below the detection limit, in contrast to Pb concentrations in roach and white bream, as also shown in Table 1. In addition, we detected Pb in the range of 0.023–0.029 µg g^−^^1^ (Table 1), while the Pb concentration in fish from VIS was below the detection limit in the study by Kostić et al. [37]. The observed concentrations of Pb in the muscle tissue of both fish species could be related to the type of feeding, as they reside at the bottom and feed on benthic organisms [40]. The Zn concentration has similar mean values to those found by Subotić et al. [39]. Nickel enrichment in this study is similar to that measured in sichel, but much higher than that in ruffe [38], which is not surprising given the fact that the concentration of Ni in aquatic ecosystems is increasing as a consequence of industrial and urban activities [41]. On the other hand, in this study, the concentrations of As, Cr and Cu in the muscles of roach and white bream at the polluted site VIS were notably lower than in white bream sampled at the same site in 2014 [37]. There is disagreement in the literature about the bioaccumulation potential of Cr and As in freshwater fish e.g., [42,43,44]. Although the data are very limited, they show that the concentrations of As and Cr and other elements vary greatly among different freshwater fish species and in different waters, although the reasons for this have not been thoroughly investigated [45].

### 3.2. Fatty Acid Composition in the Muscle Tissue

**Figure 3:**
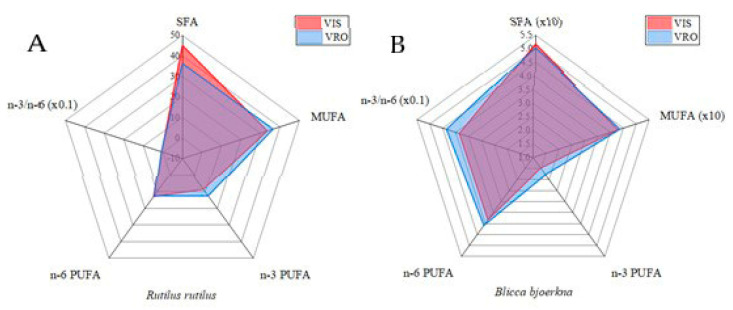
Radar plots presenting concentration of fatty acids in *roach* (**A**) and *white bream* (**B**) at the VRO and VIS localities.

Twenty-six fatty acids consisting of nine SFAs, seven MUFAs, and ten PUFAs were identified (Table 2, Figure 3). The species studied contained higher levels of SFAs than MUFAs and PUFAs at both sites, with the exception of roach from VRO, where the levels of SFAs and MUFAs were almost equal. Saturated fatty acids varied greatly among species, and only roach showed large differences among sites. Among SFAs, myristic acid (C14:0), palmitic acid (C16:0), and stearic acid (C18:0) dominated, accounting for an average of 90.4% and 94.8% of SFAs in roach and white bream, respectively. Monounsaturated fatty acid content varied spatially within each fish species (Table 2, Figure 3). The predominant MUFAs, which accounted for approximately 90.1% overall, were palmitoleic acid (C16:1) and oleic acid (C18:1) in roach and 98% in white bream (Table 2, Figure 3). The total SFA content in roach was 36.311% and 45.116% at the VRO and VIS sites, respectively, which is comparable to the values obtained in roach from Lake Gusinoe and the Olsztyn Lake District. With respect to ƩMUFA, the results were 2.5 to 3 times higher, while for ƩPUFA, an opposite trend was found than in the above studies. In bream from both localities studied, we found a much higher content of ΣSFA (Table 2), which contradicts the results of [26,46].

The percentage of PUFA was higher in both fish at the VRO site. Among PUFA, n-6 acids dominated over n-3 acids, especially C18:2. It was found that alpha-linoleic acid (ALA, 18:3, n-3), eicosapentaenoic acid (EPA, 20:5 n-3), and docosahexaenoic acid (DHA, 22:6, n-3) were present in significantly higher amounts in roach compared to white bream regardless of the sampling location. Freshwater fish have higher levels of C18 PUFA and lower levels of n-3 EPA and DHA compared to marine fish. They are also known for their high content of n-6-PUFA, especially ALA acid and arachidonic acid [47,48]. The content of fatty acids in the muscle tissue of the two fish species studied was not significantly different using a Mann–Whitney test (*p* < 0.05), but differences cannot be excluded. It is already known that differences in FA profiles between species follow taxonomic differences, and these differences are thought to be due not only to dietary differences but also to metabolic differences [49].

In addition, the ratio of n-3 and n-6 fatty acids was calculated (Table 2, Figure 3). Previous reports have shown that an optimal n-3/n-6 PUFA ratio can improve the growth performance, nutrient utilization, and health of aquatic animals [50,51,52]. In the study of Dong et al. (2023) [53], the effects of dietary n-3/n-6 polyunsaturated fatty acid (PUFA) ratio on the growth performance, lipid metabolism, hepatic antioxidant status, and gut flora of spotted seabass were explored. It is indicated that the dietary n-3/n-6 PUFA ratio may affect growth performance by regulating lipid metabolism. The extremely high and low dietary n-3/n-6 PUFA ratios induced lipid metabolism dysfunction [53]. The optimal ratio of n-6/n-3 PUFAs should be about 1–2:1 for normal physiological functions in the body due to the competitiveness of n-6 and n-3 PUFAs [54,55]. Accordingly, in our study only white bream exhibited an n-6/n-3 ratio greater than 2.1, regardless of sampling location. Roach showed a moderately elevated value of 1.16 and 1.51 at the VRO and VIS sites, respectively.

### 3.3. PCA Analysis

**Figure 4:**
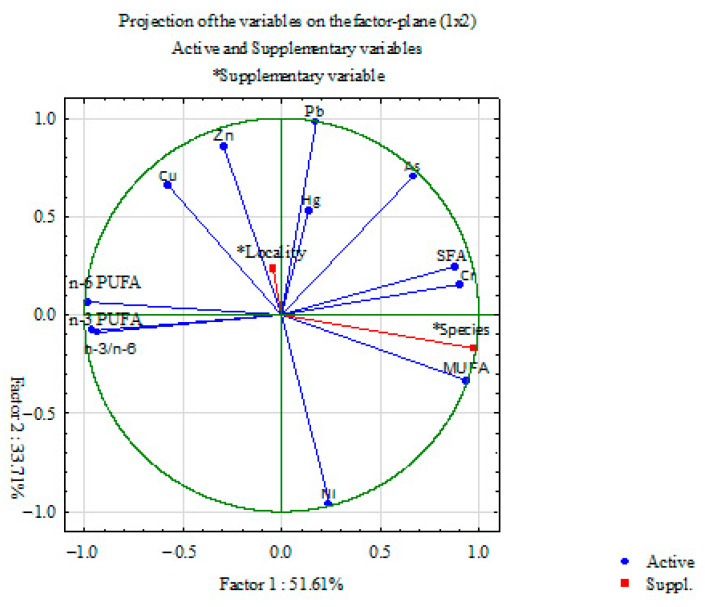
Principal component analysis (PCA) of all investigated parameters. Projection on the factor plane considering investigated roach and white bream sampled from both localities Veliko Ratno Ostrvo (VRO) and Višnjica (VIS). * Supplementary variable.

**Figure 5:**
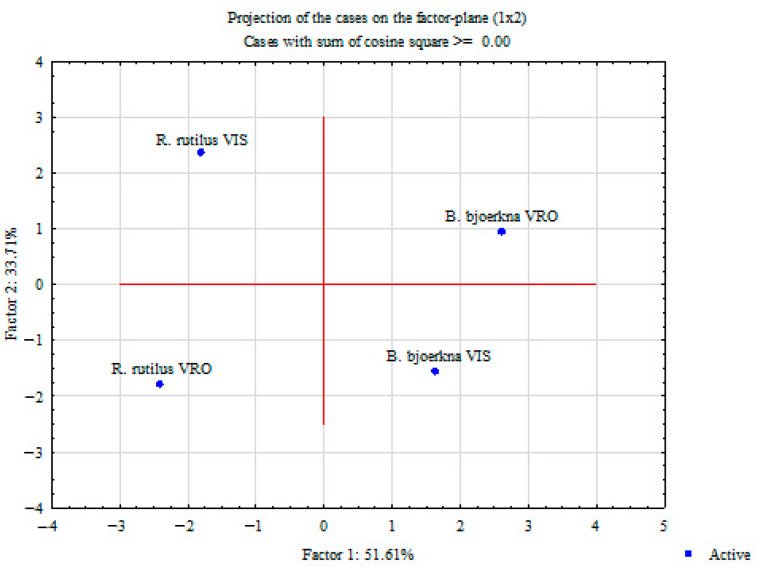
Principal component analysis (PCA) of contribution of investigated parameters based on correlations in roach and white bream from Veliko Ratno Ostrvo (VRO) and Višnjica (VIS) localities. Projection on the factor plane.

Principal component analysis was used to determine a possible separation of the two groups of fish studied based on all the parameters examined. PCA was performed in two ways: by projecting the relative contribution of each studied parameter into the factor plane (Figure 4) and by projecting the species and localities based on the studied parameters (Figure 5). PCA, which refers to the relative contribution of each parameter studied, showed that PC1 and PC2 can explain about 85.32% of the total variance in the data matrix. PC1 explained 51.61% of the total variance, with n-6 PUFA, n-3 PUFA and MUFA being the parameters contributing the most to the differences (Table 3). PC2 explains 33.71% of the total variance, with Pb, Ni and Zn being the parameters contributing most to the differences (Table 3). Summarizing the results of PCA for both species and localities, considering the parameters studied, PC1 and PC2 can explain 85.32% of the total variance. PC1 (51.61%) clearly distinguishes the studied species (roach on the left side of the y-axis and white bream on the right side). PC2 (33.71%) clearly distinguishes between VRO and VIS, and thus we cannot conclude that the localities have an influence on the investigated parameters.

### 3.4. Correlation of Metal and Trace Element Concentrations with FA Content

Spearman’s rank correlation coefficients between the analyzed fatty acids and the concentrations of metals and trace elements have shown very strong positive and negative correlations for both species (Table 4 and Table 5).

In white bream, Spearman’s rank order correlation coefficients showed significant positive correlations between locality and Cr, C13:0, C14.1, C15:0, C18:1b, and C20:1, as well as negative correlation between locality and Hg concentration (*p* < 0.05).

Interestingly, in roach, we obtained positive correlations of locality with metals only, e.g., with Cu, Pb, and Zn (*p* < 0.05). These data may indicate a different physiological response of the two fish species studied to living in two different localities, or different adaptive metabolic mechanisms as a result of different feeding behaviors. It is noticeable that in the roach, there are only correlations between the locality and some metals (Cu, Zn and Pb), and for white bream, there are correlations with metals (Cr and Hg) and with some groups of fatty acids (C13:0, C14.1, C15:0, C18:1b and C20:1), which confirms our assumption.

In white bream, strong positive correlations were found between fish length and the concentration of metals and trace elements for As and Hg, while in roach, length was negatively correlated with Zn. With respect to FA, correlations were found only for white bream, positively for C18:1a, and negatively for C13:0, C14:1, C14:1, C18:1b and C20:1 (see Supplementary Tables S3 and S4). Based on the obtained results, it can be seen that in white bream, the concentrations of metals and trace elements and composition of fatty acids in muscle can be influenced by fish size.

In the work of Fonseca et al. [23], C14:0 and C14:1 in common goby (*Pomatoschistus microps*) showed no correlation with metal concentrations, while this study’s results regarding C14:1 in white bream showed correlation with locality. In the same study, most FA had negative correlations with the measured metals, while the correlation data between metals and specific FA in this study had an almost equal number of positive and negative correlations.

In the reviewed studies with fish, a change in the content of PUFAs, MUFAs and SFAs was observed after exposure to organic and inorganic pollutants [4,56,57]. A notable observation from the results obtained was that the higher ƩSFA values were recorded in both fish species caught at VIS. Higher ƩMUFA and ƩPUFA values were recorded in both fish species caught at the VRO site, which makes fish potentially vulnerable to peroxidative attack due to the large amounts of PUFAs in their tissues [58]. The main reason for the changes in PUFAs is that they could be oxidized under oxidative stress conditions induced by metals or other compounds [59,60]. Metal ions are directly or indirectly involved in lipid peroxidation reactions and enhanced free radical formation [22,61]. Metals such as Cd, Ni and Hg can induce the formation of reactive oxygen species due to their oxidative potential, which can lead to lipid peroxidation damage [62,63]. Nickel, which has the highest bioaccumulation in roach and white bream (Table 1), can induce the formation of reactive oxygen species by binding to macromolecules and inactivating protective antioxidant enzymes [64].

## 4. Conclusions

As far as we know, the literature on this topic is very sparse, considering that most studies focus on the nutritional benefits of fish consumption for humans in terms of FA content and n-3/n-6 ratio. The results of this study show different FA profiles under various anthropogenic pressures in the Belgrade urban area, which could be attributed to the bioaccumulation of non-essential metals in the muscles of the studied fish species. The analysis of the concentration of nine elements in roach and white bream showed species-specific differences in their bioaccumulation. Although the results show that both fish species are safe from a nutritional and health point of view and the metal concentrations are below the maximum values set by national legislation, it is recommended that monitoring studies be carried out at regular intervals to investigate metal concentrations, even if the values are not above acceptable standards. This study confirmed the influence of diet on metal accumulation and indicated that FAs are very useful and complementary tools to link metal accumulation in fish with their trophic ecology.

## Figures and Tables

**Table 1:** Metal and trace element concentrations (As, Cd, Co, Cr, Cu, Hg, Pb, Zn, and Ni) in the muscle of roach (n = 16) and white bream (n = 16) (means ± standard deviation) at studied localities, Veliko Ratno Ostrvo (VRO) and Višnjica (VIS). Concentrations are expressed as µg g^−1^ of wet weight. (Mann–Whitney U test, *—significant results in comparison with upstream site—VRO, * *p* < 0.05).

Metal (µg g^−1^)	VRO		VIS	
	*R. rutilus*	*B. bjoerkna*	*R. rutilus*	*B. bjoerkna*
As	0.019 ± 0.003	0.115 ± 0.067	0.072 ± 0.016 *	0.048 ± 0.019
Cd	bdl ^#^	bdl	bdl	bdl
Co	bdl	bdl	bdl	bdl
Cr	0.019 ± 0.003	0.110 ± 0.021	0.020 ± 0.004	0.051 ± 0.013
Cu	0.264 ± 0.025	0.202 ± 0.024	0.566 ± 0.107	0.269 ± 0.019
Hg	0.080 ± 0.007	0.109 ± 0.043	0.081 ± 0.031	0.050 ± 0.029
Ni	0.480 ± 0.024	0.317 ± 0.051	0.158 ± 0.008	0.591 ± 0.054
Pb	0.023 ± 0.003	0.028 ± 0.007	0.029 ± 0.002 *	0.024 ± 0.007
Zn	9.002 ± 0.840	12.935 ± 0.760	27.641 ± 0.789 *	12.228 ± 0.773

Legend: # bdl—below detection limit.

**Table 2:** Fatty acids’ profiles (presented as percentage of total fatty acids) in muscle tissues of roach and white bream caught from Veliko Ratno Ostrvo (VRO) and Višnjica (VIS) localities. Data are expressed as mean ± SD.

Fatty Acid	VRO		VIS	
	*R. rutilus*	*B. bjoerkna*	*R. rutilus*	*B. bjoerkna*
C11:0	0.009 ± 0.008	/	0.008 ± 0.006	/
C12:0	0.592 ± 0.145	0.400 ± 0.060	0.707 ± 0.327	0.653 ± 0.271
C13:0	0.099 ± 0.027	0.082 ± 0.029	0.156 ± 0.133	0.189 ± 0.047
C14:0	6.517 ± 0.963	5.035 ± 0.530	7.545 ± 2.456	6.477 ± 1.471
C14:1	0.099 ± 0.049	0.076 ± 0.034	0.144 ± 0.093	0.133 ± 0.036
C15:0	1.381 ± 0.242	0.825 ± 0.153	1.767 ± 0.841	1.320 ± 0.328
C15:1	0.876 ± 0.172	/	1.328 ± 0.928	/
C16:0	19.255 ± 13.279	38.399 ± 2.296	25.456 ± 10.740	35.152 ± 3.605
C16:1	14.210 ± 2.222	11.379 ± 0.648	10.063 ± 7.105	11.973 ± 1.705
C17:0	0.988 ± 0.435	0.528 ± 0.185	1.346 ± 0.699	0.949 ± 0.325
C17:1	0.692 ± 0.208	/	0.928 ± 0.469	/
C18:0	7.123 ± 2.120	4.975 ± 0.461	7.711 ± 3.395	6.887 ± 1.292
C18:1	0.157 ± 0.051	27.772 ± 1.551	0.185 ± 0.090	24.905 ± 1.381
C18:1	19.360 ± 10.579	4.056 ± 0.123	19.512 ± 12.102	5.090 ± 0.832
C18:2	11.770 ± 2.241	3.556 ± 2.464	10.551 ± 1.807	3.256 ± 2.010
C18:2	/	0.090 ± 0.057	/	0.076 ± 0.012
C18:3	0.289 ± 0.054	0.231 ± 0.033	0.299 ± 0.109	0.378 ± 0.360
C18:3	1.635 ± 0.469	/	1.284 ± 0.512	/
C20:0	0.347 ± 0.144	0.181 ± 0.039	0.419 ± 0.271	0.167 ± 0.038
C20:1	1.106 ± 0.459	0.419 ± 0.063	1.402 ± 0.795	0.788 ± 0.264
C20:2	2.095 ± 0.602	0.299 ± 0.219	1.446 ± 0.474	0.394 ± 0.196
C20:3	0.593 ± 0.117	0.097 ± 0.054	0.547 ± 0.148	0.057 ± 0.046
C20:4	2.543 ± 0.885	0.313 ± 0.057	1.857 ± 0.558	0.241 ± 0.041
C20:4	/	0.048 ± 0.040	/	0.051 ± 0.019
C20:5	3.719 ± 2.439	0.603 ± 0.030	2.563 ± 1.871	0.508 ± 0.297
C22:6	4.546 ± 2.799	0.635 ± 0.087	2.774 ± 1.713	0.354 ± 0.169
ΣSFA	36.311	50.424	45.116	51.794
ΣMUFA	36.499	43.703	33.563	42.890
Σn-3 PUFA	12.443	1.782	8.478	1.481
Σn-6 PUFA	14.746	4.091	12.843	3.836
n-3/n-6	0.844	0.436	0.660	0.386

Legend: ΣSFA (saturated fatty acid), ΣMUFA (monounsaturated fatty acid), Σn-3 PUFA (polyunsaturated fatty acid). Σn-6 PUFA (polyunsaturated fatty acid).

**Table 3:** Contribution of variables based on correlations. Variables that contributed the most are marked in bold and with asterisk.

	Variable Contributions Based on Correlations		
Variable	Factor 1	Factor 2	Factor 3
As	0.071439	0.123400	0.033139
Cr	0.131525	0.006070	0.091329
Cu	0.053179	0.106799	0.135462
Hg	0.003158	0.069734	0.396445
Ni	0.009195	0.226482 *	0.015257
Pb	0.004857	0.239604 *	0.000372
Zn	0.013966	0.181396 *	0.102018
SFA	0.124518	0.014713	0.096118
MUFA	0.142051 *	0.027178	0.005849
n-3 PUFA	0.149469 *	0.001584	0.038543
n-6 PUFA	0.156076 *	0.001025	0.016599
n-3/n-6	0.140564	0.002013	0.068868

**Table 4:** Calculation results of Spearman’s rank order correlation coefficients in white bream between metals and concentration of fatty acids and investigated localities Veliko Ratno Ostrvo (VRO) and Višnjica (VIS). Only significant correlations are shown.

White Bream	Spearman’s Rank Order Correlations. MD Pairwise Deleted.		
Pair of Variables	Spearman’s R	t (N-2)	*p*-Value
Locality and Cr	0.596559	2.78122	0.014714
Locality and Hg	−0.705024	−3.71971	0.002286
Locality and C13:0	0.866025	3.87298	0.011725
Locality and C14:1	0.866025	3.87298	0.011725
Locality and C15:0	0.866025	3.87298	0.011725
Locality and C18:1b	0.866025	3.87298	0.011725
Locality and C20:1	0.866025	3.87298	0.011725

**Table 5:** Calculation results of Spearman’s rank order correlation coefficients in roach between metals and investigated localities Veliko Ratno Ostrvo (VRO) and Višnjica (VIS). Only significant correlations are shown.

Roach	Spearman’s Rank Order Correlations. MD Pairwise Deleted.		
Pair of Variables	Spearman’s R	t (N-2)	*p*-Value
Locality and Cu	0.650791	3.207135	0.006330
Locality and Pb	0.786373	4.762975	0.000303
Locality and Zn	0.813489	5.233670	0.000127

## Data Availability

All data generated or analyzed during this study are included in this article (and in the Supplementary Materials).

## Supplementary Materials

The following supporting information can be downloaded at: https://www.mdpi.com/article/10.3390/ani14060954/s1, Table S1: Number, size, and age of the two fish species sampled for each sampling site; Table S2: The operational parameters of the ICP-OES instrument (iCAP 7400 Duo Thermo Fisher Scientific); Table S3: Correlations of body length with metal and trace element concentrations in white bream and roach (*p* < 0.05). Statistically significant correlations are marked in bold and with asterisk; Table S4: Correlations of body length with fatty acids (FA) profiles in white bream (*p* < 0.05). Statistically significant correlations are marked in bold and with asterisk.
