# Supplementary material for: Effects of Trace Elements on the Fatty Acid Composition in Danubian Fish Species
Source: Animals (Basel). 2024 Mar 19;14(6):954. doi: 10.3390/ani14060954 (PMC10967273; doi:10.3390/ani14060954)
Supplement: Supplementary file 1 [file animals-14-00954-s001.zip › animals-2920323-supplementary.pdf]

animals

MDPI

Article

Effects of Trace Elements on the Fatty Acid Composition in Danubian Fish Species

Katarina Jovičić [1,*], Vesna Djikanović [1], Isidora Santrač [2], Sanja Živković [3], Milena Dimitrijević [2] and Jelena S Vranković [1]

[1] University of Belgrade, Institute for Biological Research "Siniša Stanković"—National Institute of Republic of Serbia, Department of Hydroecology and Water Protection, Bulevar despota Stefana 142, 11060 Belgrade, Serbia
[2] University of Belgrade—Institute for Multidisciplinary Research, Life Sciences Department, Kneza Višeslava 1, 11000 Belgrade, Serbia
[3] University of Belgrade, "Vinča" Institute of Nuclear Sciences—National Institute of the Republic of Serbia, Department of Physical Chemistry,11351 Belgrade, Serbia
* Correspondence: katarina.jovicic@ibiss.bg.ac.rs

Table S1. Number, size, and age of the two fish species sampled for each sampling site.

	**Veliko Ratno ostrvo**		Višnjica	
	R. rutilus	*B. bjoerkna*	*R. rutilus*	*B. bjoerkna*
**Length (cm)**				
Min – max	27.0–29.0	30.2–34.0	24.0–27.0	22.0–27.5
Average	28.05	32.25	25.3	24.87
**Weight (g)**				
Min – max	360.0–400.0	400.0–780.0	200.0–300.0	190.0–390.0
Average	380.0	607.5	247.5	267.5
Age	4^+	5^+	4^+	3^{+-} 4^+

Table S2. The operational parameters of the ICP-OES instrument (iCAP 7400 Duo Thermo Fisher Scientific).

**Parameter**	**iCAP 7400**
RF generator power	1150 W
RF generator frequency	27.12 MHz
Detector	CID86 chip
Results processing software	iTEVA iCAP
Mode for processing results	Peak height
Background correction	Manual

Table S3. Correlations of body length with metal and trace element concentrations in white bream and roach ($p < 0.05$).

	**Total length (TL)**	
**Element**	**White bream**	**Roach**
**As**	**0.711866***	−0.46131
**Cr**	−0.21371	−0.42447
**Cu**	−0.01916	−0.47753
**Hg**	**0.570377***	−0.05895
**Ni**	0.449522	−0.13265
**Pb**	0.29646	−0.33898
**⚜n**	−0.30951	**−0.65586***

* Statistically significant correlations are marked in bold and with asterisk.

Table S4. Correlations of body length with fatty acids (FA) profiles in white bream ($p < 0.05$).

FA	Total length (TL)
**C12:0**	−0.5766
**C13:0**	**0.79283***
**C14:0**	−0.50453
**C14:1**	**0.79283***
**C14:1**	**0.88292***
**C16:0**	0.738769
**C16:1**	−0.23424
**C17:0**	−0.73877
**C18:0**	−0.45047
**C18:1**	**0.918956***
**C18:1**	**−0.99103***
**C18:2**	0.162169
**18:2**	0.198206
**C18:3**	−0.10811
**C20:0**	0.324337
**C20:1**	**−0.84688***
**C20:2**	−0.39641
**C20:3**	−0.48651
**C20:4**	−0.16217
**20:4**	0.072075
**C20:5**	−0.39641
**C23:6**	0.252262

* Statistically significant correlations are marked in bold and with asterisk.
